# Nanocomposite capsules with directional, pulsed nanoparticle release

**DOI:** 10.1126/sciadv.aao3353

**Published:** 2017-12-08

**Authors:** Christiana E. Udoh, João T. Cabral, Valeria Garbin

**Affiliations:** Department of Chemical Engineering, Imperial College London, London SW7 2AZ, UK.

## Abstract

The precise spatiotemporal delivery of nanoparticles from polymeric capsules is required for applications ranging from medicine to materials science. These capsules derive key performance aspects from their overall shape and dimensions, porosity, and internal microstructure. To this effect, microfluidics provide an exceptional platform for emulsification and subsequent capsule formation. However, facile and robust approaches for nanocomposite capsule fabrication, exhibiting triggered nanoparticle release, remain elusive because of the complex coupling of polymer-nanoparticle phase behavior, diffusion, phase inversion, and directional solidification. We investigate a model system of polyelectrolyte sodium poly(styrene sulfonate) and 22-nm colloidal silica and demonstrate a robust capsule morphology diagram, achieving a range of internal morphologies, including nucleated and bicontinuous microstructures, as well as isotropic and non-isotropic external shapes. Upon dissolution in water, we find that capsules formed with either neat polymers or neat nanoparticles dissolve rapidly and isotropically, whereas bicontinuous, hierarchical, composite capsules dissolve via directional pulses of nanoparticle clusters without disrupting the scaffold, with time scales tunable from seconds to hours. The versatility, facile assembly, and response of these nanocomposite capsules thus show great promise in precision delivery.

## Teaser

Nanocomposite microcapsules with bicontinuous internal morphology release nanoparticles in directional bursts.

## INTRODUCTION

Capsules are essential vehicles for the storage and delivery of drugs, biologically active species, surfactants, and personal care formulations (*1*–*3*). The morphology of capsules has been shown to control their assembly and packing, flow, optical, magnetic, and release properties (*4*–*7*). Polymer- and nanoparticle-based capsules can be engineered to have high cargo capacity, biocompatibility, and surface functionalities underpinning growing applications in the pharmaceutical, biological, and energy industries (*8*–*10*). Various techniques, including spray drying (*11*), emulsion polymerization (*12*), solvent displacement (*13*, *14*), and layer-by-layer assembly (*15*), are used to fabricate microcapsules. Recent advances in the field of droplet microfluidics have provided an attractive platform for high-throughput, single and multiple, emulsification and templating of droplets combining exceptional size and morphology control, and low polydispersity (*16*–*18*). A range of approaches have been demonstrated for particle and capsule formation using polymerization, phase change, solvent evaporation, and directed solidification. In particular, anisotropic nanoparticle and polymer particles (*19*) have been fabricated by ultraviolet exposure (*20*), uniaxial stretching of preformed spherical particles (*21*), and solidification under flow (*4*, *22*), yielding toroidal (*22*), hollow (*23*), doughnut-shaped (*24*), and buckled (*25*) morphologies. Their internal microstructure can be spontaneously generated by demixing, coarsening, and eventual kinetic arrest upon solidification. In solvent evaporation, solute droplets are concentrated by osmotic extraction of the solvent in a liquid or gas phase (by spray drying), and this process has been used in the fabrication of spherical polymeric capsules with precise internal micropore structure and size distribution (*26*, *27*), and capsules with “bijel” (*28*) structures (*29*).

Previously, we have demonstrated the formation of spherical, neat polymer capsules in microfluidics using selective solvent extraction and achieved precise tuning of capsule size and microstructure without the use of porogens. The overall capsule formation mechanism was shown to be dependent on ternary solution thermodynamics. Here, motivated by the need for facile encapsulation and delivery of nanoparticles from capsules, with ubiquitous applications from medicine to materials science, we seek to design nanocomposite capsules with tunable morphology and microstructure, expected to nontrivially affect their dissolution behavior. Informed by the rich phase behavior (*30*) of polymer-nanoparticle mixtures, the interplay between extraction concentration pathways and solution phase boundaries, as well as competitive time scales for demixing and solidification, we develop a robust microfluidic approach for a single-stage nanocomposite capsule formation with tunable nanoparticle release.

## RESULTS

### Experimental approach

**Fig. 1:**
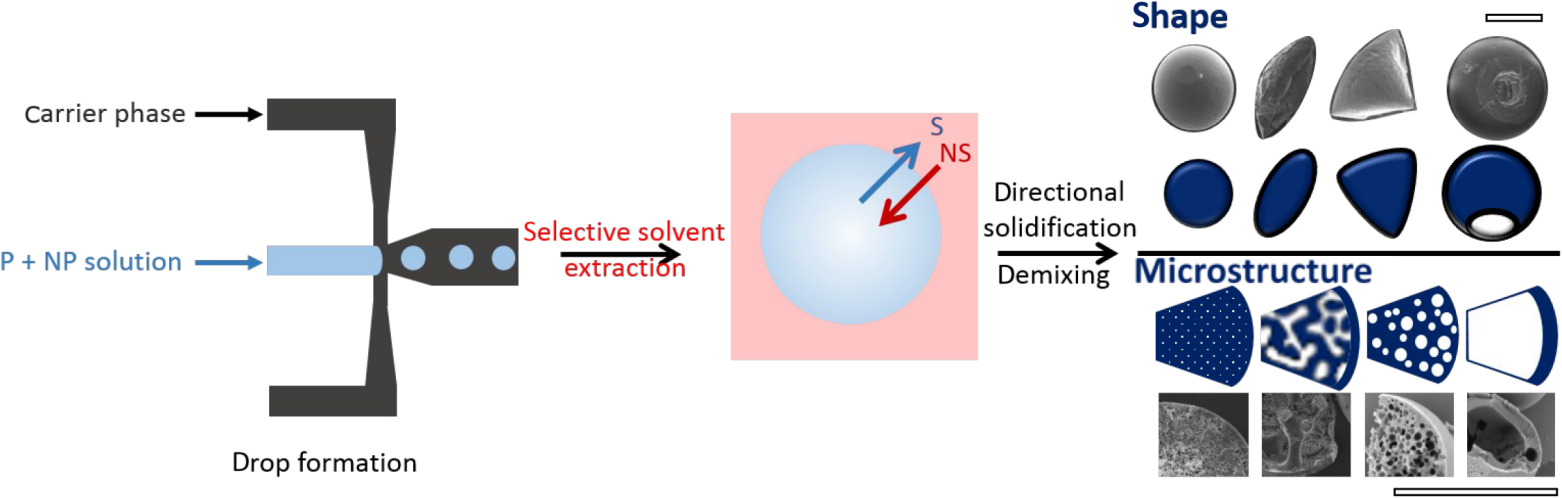
Schematic of the capsule formation process. Polymer (P)–nanoparticle (NP) solutions are emulsified into droplets in a microfluidic flow-focusing junction, followed by capsule formation via solvent extraction and directed solidification. A range of internal and external morphologies are accessible by varying drop size and (ternary) composition. For the data presented here, the polymer was NaPSS, the nanoparticles were silica (22 nm in diameter), and the solvent (S), carrier, and nonsolvent (NS) were water, hexadecane, and ethyl acetate, respectively. Scale bar, 100 μm.

We select a model system of a water-soluble polyelectrolyte [sodium poly(styrene sulfonate) (NaPSS)] and silica nanoparticles, with a diameter of 22 nm. Polymer-nanoparticle aqueous mixture droplets are first generated within an immiscible carrier phase (hexadecane) and then subjected to solvent extraction in ethyl acetate, a nonsolvent for the polymer but a good solvent for both the carrier and droplet phases. We use a flow-focusing microfluidic device, fabricated by frontal photopolymerization (FPP) (*31*, *32*), to achieve high throughput of droplet production with precise dimensions and frequency. Our approach is illustrated in Fig. 1. Upon extraction and reduction of droplet volume, the solute concentration increases (polymer or nanoparticle) and, accompanied by nonsolvent exchange at the interface, leads to phase separation by nucleation and growth or spinodal decomposition and, finally, directional solidification. The solvent interdiffusion process is akin to the phase inversion used extensively in membrane fabrication. We design the drop shrinkage to yield relatively high Péclet numbers (the ratio of the extraction rate to the diffusion coefficient of the solute), from approximately 3 to 20, such that a droplet skin is formed at an early stage of extraction (*33*). These design principles enable precise control of the shape of the capsule through shell mechanics (see schematics and scanning electron micrographs in Fig. 1) and of the internal microstructure (see Fig. 1).

### Droplet extraction, demixing, and deformation

**Fig. 2:**
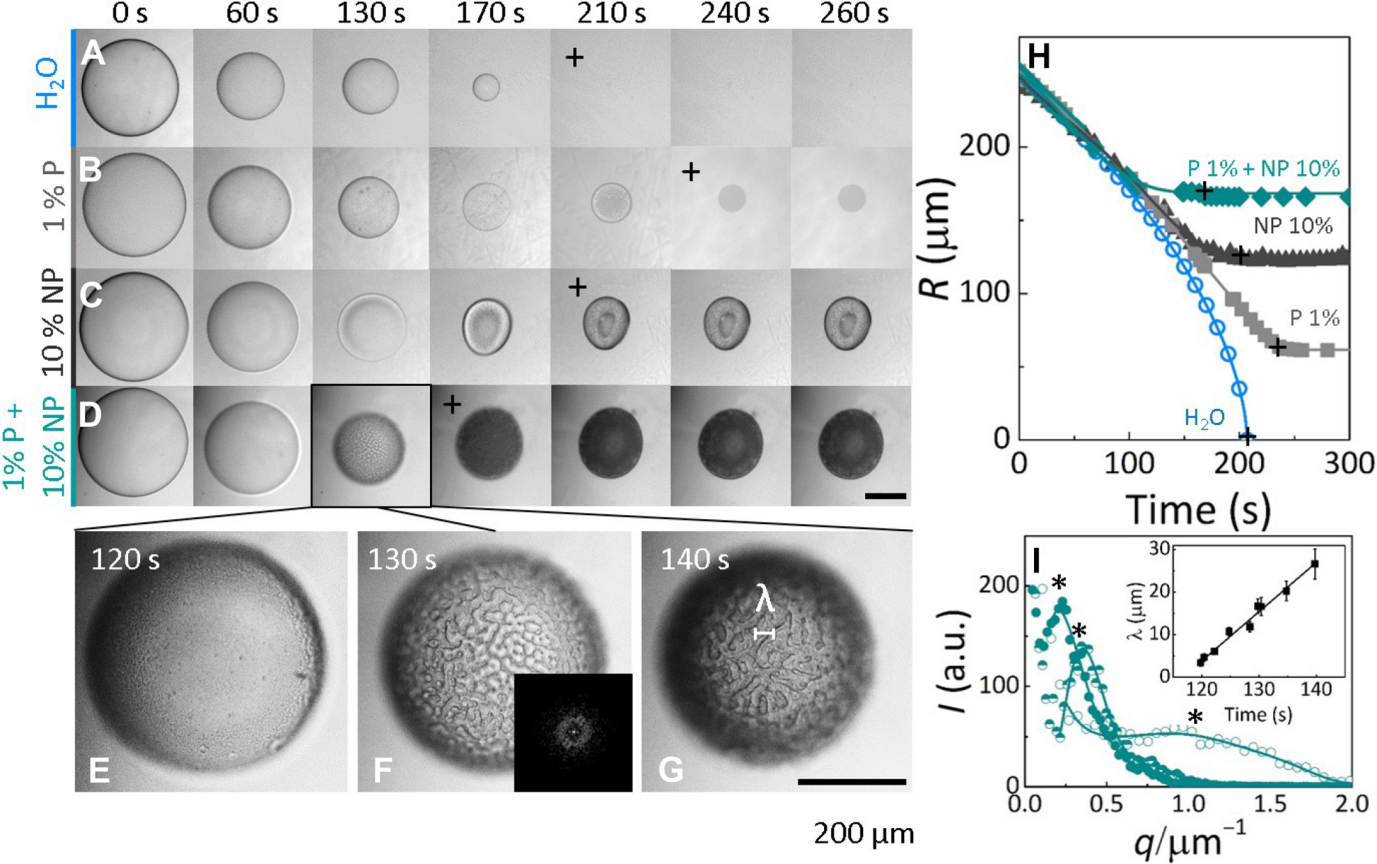
Optical images and kinetics of solvent extraction. Time series of solvent extraction from droplets of (**A**) pure H_2_O, (**B**) 1% (w/v) NaPSS (P), (**C**) 10% (w/v) silica (S), and (**D**) 1% (w/v) NaPSS + 10% (w/v) silica droplets; the initial droplet radius was *R*_0_ ≃ 250 μm. (**E** to **G**) Higher-magnification images of spinodal decomposition and coarsening of surface morphology of (D) within 120 to 140 s; inset in (F) shows corresponding FFT of the structure. (**H**) Evolution of droplet radius with time during extraction for data in (A) to (D). The symbol “+” indicates the completion of the extraction process (or dissolution for pure water). Droplets and then capsules remain approximately spherical in all cases at these compositions. Extraction kinetics for droplets with *R*_0_ ≃ 140 μm is provided in fig. S2. (**I**) Structure factor obtained from FFT of images (E to G), with the inset showing the evolution of dominant length scale (λ ≡ 2π/q*) with time, where q* is the wave number of the peak position indicated with “*”. A linear λ ∝ *t*^1^ dependence is expected for hydrodynamic coarsening in three dimensions. a.u., arbitrary units.

Mixtures of nanoparticles and polymers exhibit a rich phase behavior with coexisting regions of nanoparticle-poor “gas” and nanoparticle-rich “liquid” phases because of depletion interactions (*30*). We therefore first determine the phase behavior of the polymer-nanoparticle system in solution (fig. S1) and ensure that all initial droplet concentrations are within the one-phase region, resulting in stable suspensions free from sedimentation or aggregation. Figure 2 compares the solvent extraction of droplets with identical initial size and varying polymer (*C*_NaPSS_) and nanoparticle composition ($C_{{\text{SiO}}_{2}}$). For reference, we include the dissolution of a pure water droplet in Fig. 2A. Our first observation is that the final capsule sizes obtained from both neat polymer solutions (Fig. 2B) and neat nanoparticle suspensions (Fig. 2C) are considerably smaller than those obtained from polymer-nanoparticle solution droplets (Fig. 2C). At these concentrations, the composite capsule is up to 30 times larger in volume compared to the neat polymer capsule. During solvent extraction, demixing occurs in droplets containing polymers because of the thermodynamic incompatibility between the polymer and nonsolvent. Under these conditions, we observe phase separation by nucleation and growth for neat polymer solution droplets and spinodal decomposition in droplets with high $C_{{\text{SiO}}_{2}}$ and low *C*_NaPSS_, as shown in Fig. 2 (E to G). Neat nanoparticle droplets result in compact aggregate capsules. The extraction kinetics shown in Fig. 2H were quantified by the change in droplet radius over time; as expected, the final capsule size and, to some extent, extraction time depend on the solute content of the droplets. The initial shrinkage profile is shared by all droplet compositions, including pure water, and is governed by the solvent exchange at the interface. The lines are empirical fits to $R(t)=(R_{0}-R_{\infty }){\left(1-\frac{t}{\tau }\right)}^{\alpha }+R_{\infty }$, where *R*_0_ is the initial droplet radius, *R*_∞_ is the (final) capsule radius, τ is the extraction time (when *R* ceases to change), and α is a parameter accounting for non-Fickian diffusion, introduced previously (*26*). Although the droplets remain largely spherical during capsule formation, dimples appear at high $C_{{\text{SiO}}_{2}}$ [≥10% (w/v)]. The inset shown in Fig. 2F is the fast Fourier transform (FFT) of the spinodal structure obtained for *C*_NaPSS_ = 1 % (w/v) and $C_{{\text{SiO}}_{2}}$ = 10 % (w/v), whose corresponding structure factors are obtained from the radial average of the FFTs, as shown in Fig. 2I. The evolution of the characteristic domain size of the spinodal structure is shown in the inset of Fig. 2I and is obtained by λ = 2π/q*, where q* is the peak position. The linear scaling observed is expected for coarsening due to hydrodynamic shape relaxation, λ ≈ (γ/μ)*t*^1^, where γ is the interfacial tension between phases and μ is the viscosity (*34*). Composition fluctuations normal to the capsule surface, which would indicate surface-directed spinodal decomposition (*35*, *36*), are not observed in our experiments potentially because of convection during demixing and eventual kinetic arrest of the spinodal structure. The observed spinodal decomposition is a bulk phase phenomenon.

**Fig. 3:**
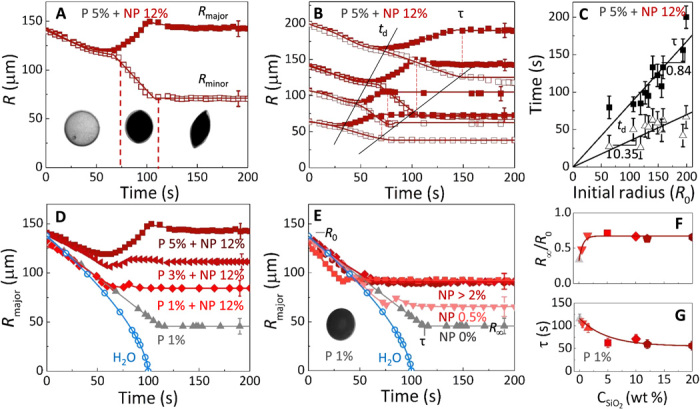
Kinetics of capsule formation for various NaPSS/silica initial compositions. (**A**) Droplet extraction kinetics for a composition of 5% (w/v) NaPSS + 12% (w/v) silica, yielding anisotropic capsules with major and minor radii indicated. Vertical dashed lines and optical images indicate the regions of liquid droplets, capsule shell formation and shape deformation, and solidification. The evolution of area and deformation [*D* = (*R*_major_ + *R*_minor_)/(*R*_major_ − *R*_minor_)] with time, for the droplet, is shown in fig. S3. (**B** and **C**) Linear dependence of final capsule size, bifurcation time, *t*_d_, and extraction time, τ, on initial droplet radius, *R*_0_, for a composition of 5% (w/v) NaPSS + 12% (w/v) silica. (**D**) Effect of polymer concentration on extraction kinetics at 12% (w/v) silica. Pure H_2_O and NaPSS 1% (w/v) are included for reference. A graph of deformation versus time for each *C*_NaPSS_ showing an increase in deformation with polymer concentration is shown in fig. S4B. (**E**) Effect of silica concentration on extraction kinetics at a constant NaPSS [1% (w/v)] concentration. Results for nanoparticle of 2 to 20% (w/v) overlap. (**F**) Ratio of final capsule size *R*_∞_ to initial droplet size *R*_0_. (**G**) Corresponding extraction time obtained in (E). Only major axis (*R*_major_) is shown in (B), (D), and (E) for clarity. Minor axis (*R*_minor_) data are shown in figs. S4A and S5.

Upon increasing polymer and nanoparticle content, significant deformation is observed during the evolution from droplet to capsule, as illustrated in Fig. 3A. The linear dependence of capsule size and extraction time, τ, on initial droplet radius, *R*_0_, is shown in Fig. 3 (B and C) for droplets with initial composition *C*_NaPSS_ = 5% (w/v) and $C_{{\text{SiO}}_{2}}$ = 12% (w/v). Because of the deviation from sphericity, the drop shrinkage kinetics are described by a major radius, *R*_major_, and minor radius, *R*_minor_, and proceed as follows. The droplet volume first decreases isotropically as solvent is extracted, whereas the droplet solution remains single-phase, and demixing takes place because of the ingress of nonsolvent and solute environment of the droplet. Polymer and nanoparticle accumulation at the interface leads to further radial concentration gradients and the eventual formation of a porous shell. Structural coarsening proceeds, and the droplet surface undulates. Strong surface enrichment leads to smooth droplet surfaces. Further volume reduction leads to the deformation of this shell, by elongation, and the creation of folds and dimples. Buckling is driven by a spontaneous reduction in compressive stresses developed on the shell wall during the reduction in volume (*37*). The deformation of the polymer-nanoparticle shell is evidently dependent on the initial *C*_NaPSS_ and $C_{{\text{SiO}}_{2}}$. Composite droplets with high polymer and silica content are found to deform by elongation and inextensional folding, with aspect ratios reaching ≈ 5, as shown in Fig. 3A, reminiscent of the folding of pollen grains (*38*). Increasing the *C*_NaPSS_ at fixed $C_{{\text{SiO}}_{2}}$ (shown in Fig. 3D) is found to enhance anisotropy such that the final major radius exceeds the initial droplet radius. By contrast, Fig. 3E shows that the effects of nanoparticle addition saturate above ≈2% (w/v) [up to 20% (w/v) studied] at constant *C*_NaPSS_ [1% (w/v)], and capsules remain largely spherical. By keeping *C*_NaPSS_ at 1% (w/v) and *R*_0_ constant, a decrease in $C_{{\text{SiO}}_{2}}$ below 10% (w/v) results in capsules with deformed shells. From Fig. 3E, we extract the dependence of *R*_∞_ and τ on the nanoparticle concentration in Fig. 3 (F and G) and find that, above a threshold $C_{{\text{SiO}}_{2}}$ (≈2% w/v), *R*_inf_ and τ remain approximately constant for droplets with similar *R*_0_.

### A morphology map for nanocomposite capsules

**Fig. 4:**
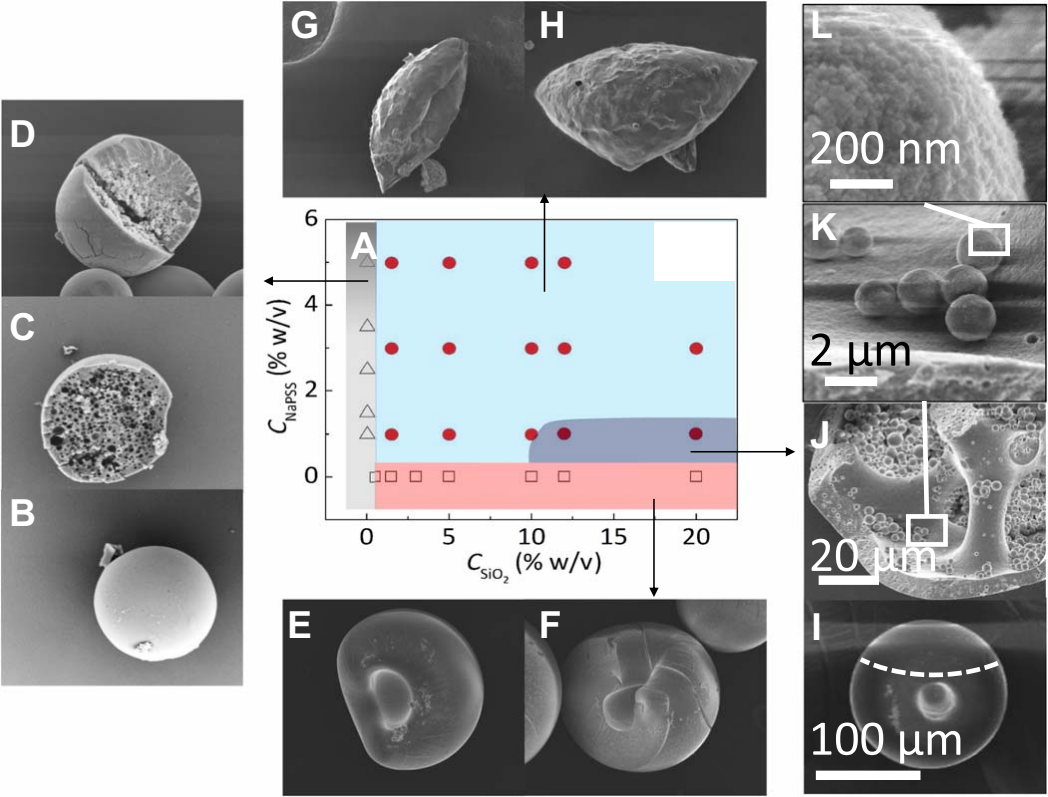
Morphology diagram. Phase map and accompanying SEM images of shape and internal structure of polymer-silica composite capsules as a function of NaPSS and SiO_2_ content for a fixed initial droplet size (*R* = 150 μm). Spherical polymer capsules with increasing size and smaller pore dimensions are obtained with increasing NaPSS concentration (Δ), shown in the gray region. Compact and dimpled capsules are obtained in the absence of polymer, with increasing size with silica content (□), shown in the pink region. At most NaPSS/SiO_2_ compositions, nonspherical capsules with folded geometries (analogous to pollen grains and tricorns) are found, shown in the light blue region. Within a narrow composition range, indicated in dark blue, dimpled capsules with a bicontinuous internal structure are obtained, comprising hierarchical silica microparticles and a composite polymer-nanoparticle shell and scaffold.

A map of the resulting capsule shape and microstructure as a function of nanoparticle and polymer content is proposed in Fig. 4A. Capsules produced from droplets of neat polymer solution, shown in Fig. 4 (C and D), exhibit smooth shells, and their internal pore size can be tuned by varying the initial *C*_NaPSS_ (*26*, *27*, *39*). Figure 4 (E and F) illustrates dense dimpled capsules produced from neat nanoparticle droplets. At most NaPSS and SiO_2_ concentrations (light blue region in Fig. 4A), elongated capsules are formed, as shown in Fig. 4 (G and H), with deformation increasing with *C*_NaPSS_ (fig. S6). Scanning electron microscopy (SEM) images reveal that the deformed capsules have thin shells (<1 μm) and are packed with ≈ 1 to 3 μm of nanoparticle clusters (figs. S7 to S9). Wide-view SEM images of the capsules from specific locations within the morphology map are shown in fig. S10. Although liquid droplets remain spherical because of the minimization of surface energy and thus area, soft shell mechanics gradually dominate as the extraction proceeds. Thin polymer films have been shown to deform primarily by stretching and bending (*40*), with the extent of deformation dependent on the ratio of stretching to bending (*24*) such that high bending energy only allows for in-plane elongation by stretching (*41*). Here, the observed soft capsules undergo extensional deformation and, eventually, experience bending deformation (out-of-plane displacement), leading to the formation of a range of pollen-grain and triangular shapes, enabling a reduction in volume (driven by osmotic drying) while retaining the surface area, imposed by skin formation. At high silica but low polymer content (shown in dark blue in Fig. 4A), dimpled capsules comprising a thin skin (≈10 μm) (Fig. 4I and fig. S9) are found, whose internal structure reveals a bicontinuous morphology (Fig. 4J), reminiscent of spinodal decomposition. The bicontinuous capsules contain hierarchical particles in the range of 1 to 3 μm (Fig. 4K), which are compact clusters of the silica nanoparticles, bound by trace amounts of the NaPSS polymer (Fig. 4L). Energy-dispersive x-ray spectroscopy (EDS) of neat and composite capsules is shown in fig. S11. A higher proportion of nanoparticles is found in the nanoparticle clusters compared to the capsule’s shell, and the compositions of the capsule scaffold and shell for the bicontinuous capsules are shown to be similar.

### Directional, pulsed release of nanoparticles upon dissolution

**Fig. 5:**
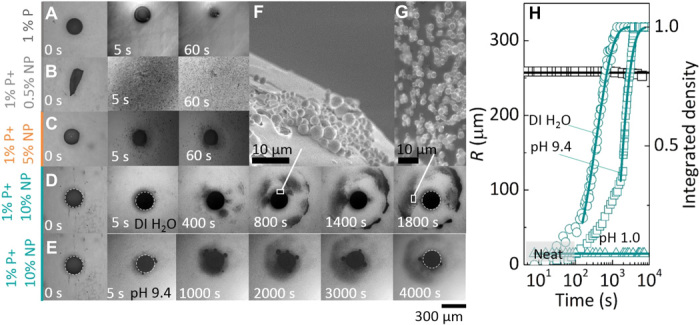
Dissolution of NaPSS/silica composite capsules. Optical images of dissolution of capsules of (**A**) 1% (w/v) NaPSS, (**B**) 1% (w/v) NaPSS + 0.5% (w/v) silica, (**C**) 1% (w/v) NaPSS + 5% (w/v) silica, and (**D**) 1% (w/v) NaPSS + 10% (w/v) SiO_2_ at pH 5 and (**E**) pH 9.4. The final capsule radius was ≃250 μm in all cases. (**F**) High-magnification images of the surface cracks of the capsule and (**G**) the emanating micrometer-sized nanoparticle clusters. (**H**) The droplet radius of the composite 1% (w/v) NaPSS/10% (w/v) SiO_2_ capsule was found to remain approximately constant over time, and the release of micrometer-sized nanoparticle clusters occurs in bursts, over long time scales, tunable with pH. Lines shown are fits of the well-known Weibull empirical model [*I* = 1 − exp(−*a*(*t* − *t*_*i*_))*^b^*, where *I* is the integrated intensity in our case; *a* and *b* are the scale and shape parameters, respectively; *t* is time; and *t*_*i*_ is the induction time] to the experimental data. DI, deionized.

The bicontinuous capsules are found to exhibit an unexpected dissolution behavior in water, which is described next. As a control, the dissolution of capsules formed from neat polymers or neat nanoparticle droplets of similar dimensions is found to take place within tens of seconds, as shown in Fig. 5A. Whereas the neat polymer capsules initially swell because of solvent ingress, the dissolution of neat nanoparticle capsules proceeds by stress cracking, as observed in the dissolution of polymer particles at low temperatures (*42*). Anisotropic composite capsules rupture upon immersion in the dissolution medium (distilled water) and release the nanoparticle clusters. The observed release is shown in Fig. 5 (B and C) for capsules produced from droplets with initial compositions of 1% (w/v) NaPSS + 0.5% (w/v) SiO_2_ and 1% (w/v) NaPSS + 5% (w/v) SiO_2_, respectively. Additional release data for anisotropic capsules produced from droplets with an initial composition of 3% (w/v) NaPSS + 10% (w/v) SiO_2_ are shown in fig. S12. By contrast, the composite capsules with a bicontinuous morphology preserve their capsule scaffold, and hence their overall shape and size, and release micrometer-sized nanoparticle clusters over a long period of time (hours) in a series of bursts, originating from specific sites at the capsule surface. The release is shown in Fig. 5D for distilled water at pH 6. The time scale of release varies markedly with pH, as illustrated in Fig. 5E, for water at pH 9.4. The ejection of the nanoparticle clusters is comparatively more diffuse under basic conditions, likely because of the higher dispersibility of the silica nanoparticles, and has a longer induction time; dissolution does not occur under strongly acidic conditions. A section of the outer shell of the capsule is shown in Fig. 5F, and an optical image of the released clusters is shown in Fig. 5G. We estimate the release in Fig. 5H by measuring the integrated density over time and find that the radius of the bicontinuous capsule remains approximately constant over time (black squares and line). The loading efficiency and extent of release of the bicontinuous capsules are estimated to be approximately 60% and 20 to 25%, respectively. Further analysis showing active sites of release at selected time periods is presented in fig. S13, and the temporal release profiles from the most active sites on the capsule surface are provided in figs. S14 and S15. Additional release data for capsules produced from higher nanoparticle payloads [15% (w/v) SiO_2_] at initial *C*_NaPSS_ [1% (w/v)] are shown in fig. S16.

## DISCUSSION

Microfluidic emulsification and droplet extraction provide an attractive route to fabricate a range of spherical and anisotropic polymer-nanoparticle capsules with controllable internal microstructure. The assembly mechanism is predicated on the engineering of the mixture thermodynamics, demixing and coarsening, phase inversion, and directional solidification during solvent extraction. We demonstrate that microporosity and capsule morphology can be precisely controlled without resorting to complex synthetic routes. The size of the capsules is primarily set by solute content and initial droplet size, and the capsule shape and morphology tuned by the polymer or nanoparticle concentration, in this case, 70,000 NaPSS and 22-nm silica. Whereas neat polymer and neat nanoparticle droplets result, respectively, in porous and compact spherical capsules, solvent extraction from droplets containing a mixture of the two components produces pollen-grain and dimpled capsules filled with clusters of nanoparticles. High nanoparticle and low polymer concentration traverse the spinodal region of this complex nanoparticle-polymer system during extraction, resulting in hierarchical capsules with bicontinuous microstructure. Surprisingly, we find that the bicontinuous capsules exhibit a unique dissolution behavior in water. Silica nanoparticles are released either as bursts or plumes, originating from specific sites on the capsule surface and spanning several hours, whereas the capsule scaffold remains intact. The time scale and profile of release can be tuned by pH, likely because of the dependence of nanoparticle dispersibility on pH. To examine the generality of the approach, we demonstrate the encapsulation of single-walled carbon nanotubes (SWCNTs) and Au nanoparticles within NaPSS capsules. Encapsulation data are shown in fig. S17 and movies S4 and S5. Because demixing between polymers and nanofillers does not take place, unlike in the silica mixture, the receding liquid-liquid interface entraps the cargo and polymer phase inversion takes place as in the case of neat polymer. As a result, the resulting composite capsules remain largely spherical at the compositions investigated. The formation of bicontinuous internal morphologies evidently requires the crossing of the spinodal line along the solvent extraction pathway, which can be engineered to match the ternary solution thermodynamics as in the case of silica-polymer mixtures, but does not readily take place. Similarly, the formation of non-isotropic capsules requires the emergence of a (composite) stiff shell during the extraction process, which deforms—by bending, folding, or crumpling—upon further volume reduction. The release of the MWCNTs/NaPSS and Au/NaPSS composite capsules is thus found to be governed by the dissolution of the polymer matrix, which occurs within time scales of minutes instead of many hours, as observed in SiO_2_/NaPSS capsules and shown in fig. S18. Overall, we find that this versatile method provides a robust route for the controlled delivery of nanofillers triggered by environmental conditions, and subject to appropriate engineering of mixture thermodynamics and liquid extraction during capsule formation, a wide range of external and internal morphologies can be attained. Further, the pulsed release mechanism can be potentially exploited in the design of asymmetric self-propelling capsules.

## METHODS

### Materials

NaPSS with an average molecular weight of 70,000, 22-nm-diameter silica nanoparticles [Ludox AS-40 nanoparticle silica; 40% (w/v) suspension in H_2_O, pH 9 to 9.5], *n*-hexadecane (ReagentPlus, ≥99%), toluene, octadecyltrichlorosilane (OTS), sorbitan monooleate (Span 80), and spherical gold (Au) nanoparticles [core radius, ≈2.3 nm; 2% (w/v) suspension in H_2_O] functionalized with the capping ligand mercaptoundecyl tetra(ethylene glycol) were obtained from Sigma-Aldrich. The capping ligand is uncharged and provides stability to the colloidal suspension by short-range steric repulsion. COOH-functionalized, multi-walled carbon nanotubes (MWCNTs) with an outer diameter of 30 to 40 nm and a length of 10 to 20 µm were obtained from Arkema. Ethyl acetate (HiPerSolv CHROMANORM, ≥99.8% purity), acetone, ethanol, and isopropyl alcohol (all AnalaR NORMAPUR) were obtained from VWR International. NOA 81 (thiolene-based prepolymer) was obtained from Norland Products, and deionized water was obtained from a Centra ELGA filtration system. All reagents were used as received.

### Phase mapping and viscosity of NaPSS/silica mixtures

Phase diagrams were estimated by turbidity 2 weeks after sample preparation to determine the thermodynamic compositional stability of the NaPSS/silica system. Colloidal silica suspensions from 1 to 40% (w/v) were prepared in weight by volume terms. Polymer was added, in 0.1-g increments, to aliquots of the prepared nanoparticle suspensions, agitated to ensure mixing, and allowed to equilibrate at 21 ± 2°C for 2 weeks. In total, more than 100 samples of different compositions were used to locate the phase boundaries with ±5% precision. One-phase (solid or gas), two-phase (solid-gas), and coexisting three-phase (solid-liquid-gas) regions of NaPSS/SiO_2_/H_2_O systems were observed by optical microscopy and visual inspection. The viscosity of NaPSS/SiO_2_/H_2_O mixtures, with a SiO_2_ concentration of 1 to 20% (w/v), was measured using a Brookfield DV-I Prime viscometer fitted with an ultralow adapter. The spindle speed was varied between 4 and 100 rpm depending on the solution concentration. All samples studied exhibit Newtonian behavior in this range, and the data are shown in table S1.

### Microfluidics and solvent extraction

A microfluidic device with flow-focusing junction was fabricated by FPP of a thiolene optical adhesive (Norland Products, NOA 81), using a previously reported procedure (*31*, *32*). The microchannels were 100 μm deep and 650 μm wide, with a focusing constriction of 300 μm. Channel surfaces were rendered hydrophobic by treating with a 10% (w/v) solution of OTS in toluene for 1 hour, followed by 24 hours in a convection oven at 110°C. Inlets were connected with silicone tubing to 10-ml syringes mounted on syringe pumps (Braintree, BS-8000), and the outlet tube was immersed in the nonsolvent bath. The dispersed phase was the polymer-nanoparticle aqueous mixture, whereas the continuous phase was hexadecane. For microfluidic emulsification, 2 to 5% (v/v) of Span 80 was used to minimize coalescence and found to have a minimal impact in capsule formation. Initial droplet radius was varied by changing the flow rate of the continuous phase, *F*_c_, within 50 to 90 μl/min, whereas the dispersed phase flow rate, *F*_d_, was kept constant at 10 μl/min, corresponding to Reynolds number (*Re* = ρ*UL*/η, where ρ is the density, *U* is the flow velocity, *L* is the characteristic length, and η is the viscosity) between 0.17 and 0.3. The polymer solution droplets, suspended in hexadecane, were then precipitated into an external nonsolvent bath of ethyl acetate in great excess volume (20 ml).

### Capsule characterization

The droplet shrinkage and evolution of internal morphology during solvent extraction were monitored using an upright reflection microscope (Olympus BX41M) and charge-coupled device camera (Allied Vision Technologies, Manta F-145, 1392 × 1040 pixels, 20 frames per second). The internal structure of the final polymer capsules was observed by SEM with a LEO 1525 field emission scanning electron microscope at an operating voltage of 5 kV, which allowed for ultrahigh resolution imaging and EDS. Capsules were dried for 24 hours, sectioned or crushed between glass plates, and coated with chromium before SEM imaging. The capsule samples were then analyzed by an energy-dispersive spectroscope (x-act, Oxford Instruments) fitted with a silicon drift detector to identify spatial distribution of polymer and nanoparticles within the capsules. X-ray intensities in counts per second were set at 100, and the accelerating voltage was set at 20 kV.

## Supplementary Material

http://advances.sciencemag.org/cgi/content/full/3/12/eaao3353/DC1

## SUPPLEMENTARY MATERIALS

Supplementary material for this article is available at http://advances.sciencemag.org/cgi/content/full/3/12/eaao3353/DC1 fig. S1. Phase diagram of NaPSS/SiO_2_/H_2_O mixtures. fig. S2. Kinetics of composite capsule formation at initial *C*_NaPSS_, 1% (w/v). fig. S3. Deformation and area change of capsule obtained from 5% (w/v) NaPSS + 12% (w/v) silica droplet. fig. S4. Kinetics of composite capsule formation at initial $C_{{\text{SiO}}_{2}}$, 12% (w/v). fig. S5. Corresponding minor axis *R*_minor_ data for Fig. 2C (main text). fig. S6. Evolution of droplet radius and deformation parameter with time during extraction for various NaPSS/silica compositions. fig. S7. SEM showing the morphology of anisotropic composite NaPSS/silica capsule. fig. S8. SEM images showing the morphology and internal microstructure of polymer-nanoparticle capsules as a function of NaPSS and SiO_2_ content. fig. S9. High-magnification SEM images showing the internal morphology of neat polymer and composite polymer-nanoparticle capsules as a function of NaPSS and SiO_2_ content. fig. S10. Wide-view SEM images showing neat polymer and composite polymer-nanoparticle capsules as a function of NaPSS and SiO_2_ content. fig. S11. EDS analysis of neat and composite NaPSS/silica capsules. fig. S12. Dissolution of 3% (w/v) NaPSS and 3% (w/v) NaPSS + 10% (w/v) SiO_2_ capsules. fig. S13. Pulsatile release of nanoparticle clusters from selected active sites of a capsule obtained from 1% (w/v) NaPSS + 10% (w/v) SiO_2_ initial droplet composition. fig. S14. Pulsatile release of nanoparticle clusters from specific sectors of capsules obtained from 1% (w/v) NaPSS + 10% (w/v) SiO_2_ and 1% (w/v) NaPSS + 15% (w/v) SiO_2_ initial droplet composition. fig. S15. Analysis of release of nanoparticle clusters from a capsule obtained from 1% (w/v) NaPSS + 10% (w/v) SiO_2_ initial droplet composition. fig. S16. Analysis of release of nanoparticle clusters from a capsule obtained from 1% (w/v) NaPSS + 15% (w/v) SiO_2_ initial droplet composition. fig. S17. Formation and SEM images of internal microstructure of composite NaPSS/SWCNT and NaPSS/Au capsules. fig. S18. Release of SWCNT and Au nanoparticles from composite NaPSS/SWCNT and NaPSS/Au capsules. table S1. Viscosity measurements of NaPSS/SiO_2_/H_2_O mixtures. note S1. Microfluidic device fabrication. note S2. Phase diagram of NaPSS/SiO_2_/H_2_O mixtures. note S3. Estimation of Péclet number. note S4. Additional analysis for data shown in the main text. note S5. Additional SEM images of capsules. note S6. EDS of neat and composite NaPSS/silica capsules. note S7. Analysis of dissolution of neat and composite NaPSS/silica capsules immersed in deionized water (pH 5 to 6). note S8. Spatiotemporal analysis of pulse release of nanoparticle clusters from bicontinuous capsules immersed in deionized water (pH 5 to 6). note S9. Impact of payload type on composite capsule morphology and release profile. movie S1. Video depicting the mechanism and kinetics of solvent extraction of 1% (w/v) NaPSS + 10% (w/v) SiO_2_ aqueous droplet of 250 μm radius in neat ethyl acetate over a period of 360 s (video 10 times faster). movie S2. Video showing the immersion of bicontinuous capsule above in deionized water (pH 5 to 6), depicting the pulsed release of nanoparticle clusters, over a time scale of 10,000 s (video 300 times faster). movie S3. As above, following immersion in deionized water with pH 9.35 over a time scale of 7000 s (video 200 times faster; air bubbles result from the immersion of the dried capsule in water and are not a result of dissolution). movie S4. Video depicting the mechanism and kinetics of solvent extraction of 1% (w/v) NaPSS + 0.3% (w/v) SWCNT aqueous droplet of ≃250 μm radius in neat ethyl acetate over a period of 250 s (video 25 times faster). movie S5. Video depicting the mechanism and kinetics of solvent extraction of 1% (w/v) NaPSS + 0.025% (w/v) Au nanoparticle aqueous droplet of ≃250 μm radius in neat ethyl acetate over a period of 250 s (video 10 times faster).

## References

[1] E. Kumacheva, P. Garstecki, in *Microfluidic Reactors for Polymer Particles* (John Wiley & Sons Inc., 2011), pp. 1–6.

[2] Langer R., Drug delivery and targeting. Nature 392, 5–10 (1998). 9579855

[3] Lim F., Sun A. M., Microencapsulated islets as bioartificial endocrine pancreas. Science 210, 908–910 (1980). 6776628 10.1126/science.6776628

[4] Velev O. D., Lenhoff A. M., Kaler E. W., A class of microstructured particles through colloidal crystallization. Science 287, 2240–2243 (2000). 10731141 10.1126/science.287.5461.2240

[5] Graf C., van Blaaderen A., Metallodielectric colloidal core-shell particles for photonic applications. Langmuir 18, 524–534 (2002).

[6] Zou R. B., Yu A. B., Evaluation of the packing characteristics of mono-sized non-spherical particles. Powder Technol. 88, 71–79 (1996).

[7] Champion J. A., Katare Y. K., Mitragotri S., Particle shape: A new design Parameter for micro- and nanoscale drug delivery carriers. J. Control. Release 121, 3–9 (2007). 17544538 10.1016/j.jconrel.2007.03.022 PMC4009069

[8] Tkachenko A. G., Xie H., Coleman D., Glomm W., Ryan J., Anderson M. F., Franzen S., Feldheim D. L., Multifunctional gold nanoparticle–peptide complexes for nuclear targeting. J. Am. Chem. Soc. 125, 4700–4701 (2003). 12696875 10.1021/ja0296935

[9] Jain P. K., Lee K. S., El-Sayed I. H., El-Sayed M. A., Calculated absorption and scattering properties of gold nanoparticles of different size, shape, and composition: Applications in biological imaging and biomedicine. J. Phys. Chem. B 110, 7238–7248 (2006). 16599493 10.1021/jp057170o

[10] Wang L., Yan R., Huo Z., Wang L., Zeng J., Bao J., Wang X., Peng Q., Li Y., Fluorescence resonant energy transfer biosensor based on upconversion-luminescent nanoparticles. Angew. Chem. Int. Ed. 44, 6054–6057 (2005). 10.1002/anie.200501907 16118828

[11] Vehring R., Foss W. R., Lechuga-Ballesteros D., Particle formation in spray drying. J. Aerosol Sci. 38, 728–746 (2007).

[12] Crespy D., Stark M., Hoffmann-Richter C., Ziener U., Landfester K., Polymeric nanoreactors for hydrophilic reagents synthesized by interfacial polycondensation on miniemulsion droplets. Macromolecules 40, 3122–3135 (2007).

[13] Fessi H., Puisieux F., Devissaguet J. Ph., Ammoury N., Benita S., Nanocapsule formation by interfacial polymer deposition following solvent displacement. Int. J. Pharm. 55, R1–R4 (1989).

[14] Freitas S., Merkle H. P., Gander B., Microencapsulation by solvent extraction/evaporation: Reviewing the state of the art of microsphere preparation process technology. J. Control. Release 102, 313–332 (2005). 15653154 10.1016/j.jconrel.2004.10.015

[15] Caruso F., Hollow capsule processing through colloidal templating and self-assembly. Chem. Eur. J. 6, 413–419 (2000). 10747405 10.1002/(sici)1521-3765(20000204)6:3<413::aid-chem413>3.0.co;2-9

[16] Xu S., Nie Z., Seo M., Lewis P., Kumacheva E., Stone H. A., Garstecki P., Weibel D. B., Gitlin I., Whitesides G. M., Generation of monodisperse particles by using microfluidics: Control over size, shape, and composition. Angew. Chem. Int. Ed. 44, 724–728 (2005). 10.1002/anie.200462226 15612064

[17] Wan J., Bick A., Sullivan M., Stone H. A., Controllable microfluidic production of microbubbles in water-in-oil emulsions and the formation of porous microparticles. Adv. Mater. 20, 3314–3318 (2008).

[18] Xu Q., Hashimoto M., Dang T. T., Hoare T., Kohane D. S., Whitesides G. M., Langer R., Anderson D. G., Preparation of monodisperse biodegradable polymer microparticles using a microfluidic flow-focusing device for controlled drug delivery. Small 5, 1575–1581 (2009). 19296563 10.1002/smll.200801855 PMC2789598

[19] Shum H. C., Abate A. R., Lee D., Studart A. R., Wang B., Chen C. H., Thiele J., Shah R. K., Krummel A., Weitz D. A., Droplet microfluidics for fabrication of non-spherical particles. Macromol. Rapid Commun. 31, 108–118 (2010). 21590882 10.1002/marc.200900590

[20] Dendukuri D., Pregibon D. C., Collins J., Hatton T. A., Doyle P. S., Continuous-flow lithography for high-throughput microparticle synthesis. Nat. Mater. 5, 365–369 (2006). 16604080 10.1038/nmat1617

[21] Crassous J. J., Mihut A. M., Månssona L. K., Schurtenberger P., Anisotropic responsive microgels with tuneable shape and interactions. Nanoscale 7, 15971–15982 (2015). 26367504 10.1039/c5nr03827h

[22] Wang B., Shum H. C., Weitz D. A., Fabrication of monodisperse toroidal particles by polymer solidification in microfluidics. Chemphyschem 10, 641–645 (2009). 19185032 10.1002/cphc.200800786

[23] Hyuk Im S., Jeong U., Xia Y., Polymer hollow particles with controllable holes in their surfaces. Nat. Mater. 4, 671–675 (2005). 16086022 10.1038/nmat1448

[24] Biswas P., Sen D., Mazumder S., Basak C. B., Doshi P., Temperature mediated morphological transition during drying of spray colloidal droplets. Langmuir 32, 2464–2473 (2016). 26900937 10.1021/acs.langmuir.5b04171

[25] Quilliet C., Zoldesi C., Riera C., van Blaaderen A., Imhof A., Anisotropic colloids through non-trivial buckling. Eur. Phys. J. E 27, 13–20 (2008). 19230134 10.1140/epje/i2007-10365-2

[26] Watanabe T., Lopez C. G., Douglas J. F., Ono T., Cabral J. T., Microfluidic approach to the formation of internally porous polymer particles by solvent extraction. Langmuir 30, 2470–2479 (2014). 24568261 10.1021/la404506b

[27] Udoh C. E., Garbin V., Cabral J. T., Microporous polymer particles via phase inversion in microfluidics: Impact of nonsolvent quality. Langmuir 32, 8131–8140 (2016). 27448632 10.1021/acs.langmuir.6b01799

[28] Herzig E. M., White K. A., Schofield A. B., Poon W. C. K., Clegg P. S., Bicontinuous emulsions stabilized solely by colloidal particles. Nat. Mater. 6, 966–971 (2007). 17982465 10.1038/nmat2055

[29] Haase M. F., Stebe K. J., Lee D., Continuous fabrication of hierarchical and asymmetric bijel microparticles, fibers, and membranes by solvent transfer-induced phase separation (STRIPS). Adv. Mater. 27, 7065–7071 (2015). 26437299 10.1002/adma.201503509

[30] Lekkerkerker H. N. W., Poon W. C.-K., Pusey P. N., Stroobants A., Warren P. B., Phase behaviour of colloid + polymer mixtures. Europhys. Lett. 20, 559–564 (1992).

[31] Cabral J. T., Hudson S. D., Harrison C., Douglas J. F., Frontal photopolymerization for microfluidic applications. Langmuir 20, 10020–10029 (2004). 15518489 10.1021/la049501e

[32] Harrison C., Cabral J. T., Stafford C. M., Karim A., Amis E. J., A rapid prototyping technique for the fabrication of solvent-resistant structures. J. Micromech. Microeng. 14, 153–158 (2004).

[33] Gundabala V. R., Zimmerman W. B., Routh A. F., A model for surfactant distribution in latex coatings. Langmuir 20, 8721–8727 (2004). 15379498 10.1021/la048939b

[34] M. Doi, Dynamics of domains and textures, in *Theoretical Challenges in the Dynamics of Complex Fluids*, T. C. McLeish, Ed. (Springer, 1997), pp. 293–314.

[35] Jones R. A. L., Norton L. J., Kramer E. J., Bates F. S., Wiltzius P., Surface-directed spinodal decomposition. Phys. Rev. Lett. 66, 1326–1329 (1991). 10043177 10.1103/PhysRevLett.66.1326

[36] Tanaka H., Wetting dynamics in a confined symmetric binary mixture undergoing phase separation. Phys. Rev. Lett. 70, 2770–2773 (1993). 10053648 10.1103/PhysRevLett.70.2770

[37] Datta S. S., Shum H. C., Weitz D. A., Controlled buckling and crumpling of nanoparticle-coated droplets. Langmuir 26, 18612–18616 (2010). 21086995 10.1021/la103874z

[38] Katifori E., Alben S., Cerda E., Nelson D. R., Dumais J., Foldable structures and the natural design of pollen grains. Proc. Natl. Acad. Sci. U.S.A. 107, 7635–7639 (2010). 20404200 10.1073/pnas.0911223107 PMC2867878

[39] Yang J., Katagiri D., Mao S., Zeng H., Nakajima H., Uchiyama K., Generation of controlled monodisperse porous polymer particles by dipped inkjet injection. RSC Adv. 5, 7297–7303 (2015).

[40] Paulsen J. D., Démery V., Santangelo C. D., Russell T. P., Davidovitch B., Menon N., Optimal wrapping of liquid droplets with ultrathin sheets. Nat. Mater. 14, 1206–1209 (2015). 26322716 10.1038/nmat4397

[41] Pauchard L., Couder Y., Invagination during the collapse of an inhomogeneous spheroidal shell. Europhys. Lett. 66, 667–673 (2004).

[42] K. Ueberreiter, The solution process, in *Diffusion in Polymers*, J. Crank, G. S. Park, Eds. (Academic Press, 1968), pp. 220–258.

