# Supplementary material for: Nanocomposite capsules with directional, pulsed nanoparticle release
Source: Sci Adv. 2017 Dec 8;3(12):eaao3353. doi: 10.1126/sciadv.aao3353 (PMC5725263; doi:10.1126/sciadv.aao3353)
Supplement: http://advances.sciencemag.org/cgi/content/full/3/12/eaao3353/DC1 [file supp_3_12_eaao3353__index.html]

Science Advances | Science Advances

## Supplementary Materials

**This PDF file includes:**

- fig. S1. Phase diagram of NaPSS/SiO2/H2O mixtures.
- fig. S2. Kinetics of composite capsule formation at initial *C*NaPSS, 1% (w/v).
- fig. S3. Deformation and area change of capsule obtained from 5% (w/v) NaPSS + 12% (w/v) silica droplet.
- fig. S4. Kinetics of composite capsule formation at initial *C*SiO2 , 12% (w/v).
- fig. S5. Corresponding minor axis *R*minor data for Fig. 2C (main text).
- fig. S6. Evolution of droplet radius and deformation parameter with time during extraction for various NaPSS/silica compositions.
- fig. S7. SEM showing the morphology of anisotropic composite NaPSS/silica capsule.
- fig. S8. SEM images showing the morphology and internal microstructure of polymer-nanoparticle capsules as a function of NaPSS and SiO2 content.
- fig. S9. High-magnification SEM images showing the internal morphology of neat polymer and composite polymer-nanoparticle capsules as a function of NaPSS and SiO2 content.
- fig. S10. Wide-view SEM images showing neat polymer and composite polymernanoparticle capsules as a function of NaPSS and SiO2 content.
- fig. S11. EDS analysis of neat and composite NaPSS/silica capsules.
- fig. S12. Dissolution of 3% (w/v) NaPSS and 3% (w/v) NaPSS + 10% (w/v) SiO2 capsules.
- fig. S13. Pulsatile release of nanoparticle clusters from selected active sites of a capsule obtained from 1% (w/v) NaPSS + 10% (w/v) SiO2 initial droplet composition.
- fig. S14. Pulsatile release of nanoparticle clusters from specific sectors of capsules obtained from 1% (w/v) NaPSS + 10% (w/v) SiO2 and 1% (w/v) NaPSS + 15% (w/v) SiO2 initial droplet composition.
- fig. S15. Analysis of release of nanoparticle clusters from a capsule obtained from 1% (w/v) NaPSS + 10% (w/v) SiO2 initial droplet composition.
- fig. S16. Analysis of release of nanoparticle clusters from a capsule obtained from 1% (w/v) NaPSS + 15% (w/v) SiO2 initial droplet composition.
- fig. S17. Formation and SEM images of internal microstructure of composite NaPSS/SWCNT and NaPSS/Au capsules.
- fig. S18. Release of SWCNT and Au nanoparticles from composite NaPSS/SWCNT and NaPSS/Au capsules.
- table S1. Viscosity measurements of NaPSS/SiO2/H2O mixtures.
- note S1. Microfluidic device fabrication.
- note S2. Phase diagram of NaPSS/SiO2/H2O mixtures.
- note S3. Estimation of Péclet number.
- note S4. Additional analysis for data shown in the main text.
- note S5. Additional SEM images of capsules.
- note S6. EDS of neat and composite NaPSS/silica capsules.
- note S7. Analysis of dissolution of neat and composite NaPSS/silica capsules immersed in deionized water (pH 5 to 6).
- note S8. Spatiotemporal analysis of pulse release of nanoparticle clusters from bicontinuous capsules immersed in deionized water (pH 5 to 6).
- note S9. Impact of payload type on composite capsule morphology and release profile.

Download PDF

**Other Supplementary Material for this manuscript includes the following:**

- movie S1 (.mp4 format). Video depicting the mechanism and kinetics of solvent extraction of 1% (w/v) NaPSS + 10% (w/v) SiO2 aqueous droplet of 250 μm radius in neat ethyl acetate over a period of 360 s (video 10 times faster).
- movie S2 (.avi format). Video showing the immersion of bicontinuous capsule above in deionized water (pH 5 to 6), depicting the pulsed release of nanoparticle clusters, over a time scale of 10,000 s (video 300 times faster).
- movie S3 (.avi format). As above, following immersion in deionized water with pH 9.35 over a time scale of 7000 s (video 200 times faster; air bubbles result from the immersion of the dried capsule in water and are not a result of dissolution).
- movie S4 (.mp4 format). Video depicting the mechanism and kinetics of solvent extraction of 1% (w/v) NaPSS + 0.3% (w/v) SWCNT aqueous droplet of ≃250 μm radius in neat ethyl acetate over a period of 250 s (video 25 times faster).
- movie S5 (.mp4 format). Video depicting the mechanism and kinetics of solvent extraction of 1% (w/v) NaPSS + 0.025% (w/v) Au nanoparticle aqueous droplet of ≃250 μm radius in neat ethyl acetate over a period of 250 s (video 10 times faster).

**Files in this Data Supplement:**

- Adobe PDF - aao3353\_SM.pdf
