# Supplementary material for: Nanocomposite capsules with directional, pulsed nanoparticle release
Source: Sci Adv. 2017 Dec 8;3(12):eaao3353. doi: 10.1126/sciadv.aao3353 (PMC5725263; doi:10.1126/sciadv.aao3353)
Supplement: http://advances.sciencemag.org/cgi/content/full/3/12/eaao3353/DC1 [file aao3353_SM.pdf]

Science Advances

AAAS

advances.sciencemag.org/cgi/content/full/3/12/eaao3353/DC1

Supplementary Materials for

Nanocomposite capsules with directional, pulsed nanoparticle release

Christiana E. Udoh, João T. Cabral, Valeria Garbin

Published 8 December 2017, *Sci. Adv.* **3**, eaao3353 (2017)
DOI: 10.1126/sciadv.aao3353

The PDF file includes:

- fig. S1. Phase diagram of NaPSS/SiO_2/H_2O mixtures.
- fig. S2. Kinetics of composite capsule formation at initial C_{NaPSS}, 1% (w/v).
- fig. S3. Deformation and area change of capsule obtained from 5% (w/v) NaPSS + 12% (w/v) silica droplet.
- fig. S4. Kinetics of composite capsule formation at initial C_{SiO_2}, 12% (w/v).
- fig. S5. Corresponding minor axis R_{minor} data for Fig. 2C (main text).
- fig. S6. Evolution of droplet radius and deformation parameter with time during extraction for various NaPSS/silica compositions.
- fig. S7. SEM showing the morphology of anisotropic composite NaPSS/silica capsule.
- fig. S8. SEM images showing the morphology and internal microstructure of polymer-nanoparticle capsules as a function of NaPSS and SiO_2 content.
- fig. S9. High-magnification SEM images showing the internal morphology of neat polymer and composite polymer-nanoparticle capsules as a function of NaPSS and SiO_2 content.
- fig. S10. Wide-view SEM images showing neat polymer and composite polymer-nanoparticle capsules as a function of NaPSS and SiO_2 content.
- fig. S11. EDS analysis of neat and composite NaPSS/silica capsules.
- fig. S12. Dissolution of 3% (w/v) NaPSS and 3% (w/v) NaPSS + 10% (w/v) SiO_2 capsules.
- fig. S13. Pulsatile release of nanoparticle clusters from selected active sites of a capsule obtained from 1% (w/v) NaPSS + 10% (w/v) SiO_2 initial droplet composition.
- fig. S14. Pulsatile release of nanoparticle clusters from specific sectors of capsules obtained from 1% (w/v) NaPSS + 10% (w/v) SiO_2 and 1% (w/v) NaPSS + 15% (w/v) SiO_2 initial droplet composition.

- fig. S15. Analysis of release of nanoparticle clusters from a capsule obtained from 1% (w/v) NaPSS + 10% (w/v) SiO_2 initial droplet composition.
- fig. S16. Analysis of release of nanoparticle clusters from a capsule obtained from 1% (w/v) NaPSS + 15% (w/v) SiO_2 initial droplet composition.
- fig. S17. Formation and SEM images of internal microstructure of composite NaPSS/SWCNT and NaPSS/Au capsules.
- fig. S18. Release of SWCNT and Au nanoparticles from composite NaPSS/SWCNT and NaPSS/Au capsules.
- table S1. Viscosity measurements of NaPSS/SiO_2/H_2O mixtures.
- note S1. Microfluidic device fabrication.
- note S2. Phase diagram of NaPSS/SiO_2/H_2O mixtures.
- note S3. Estimation of Péclet number.
- note S4. Additional analysis for data shown in the main text.
- note S5. Additional SEM images of capsules.
- note S6. EDS of neat and composite NaPSS/silica capsules.
- note S7. Analysis of dissolution of neat and composite NaPSS/silica capsules immersed in deionized water (pH 5 to 6).
- note S8. Spatiotemporal analysis of pulse release of nanoparticle clusters from bicontinuous capsules immersed in deionized water (pH 5 to 6).
- note S9. Impact of payload type on composite capsule morphology and release profile.

Other Supplementary Material for this manuscript includes the following:
(available at advances.sciencemag.org/cgi/content/full/3/12/eaao3353/DC1)

- movie S1 (.mp4 format). Video depicting the mechanism and kinetics of solvent extraction of 1% (w/v) NaPSS + 10% (w/v) SiO_2 aqueous droplet of 250 μm radius in neat ethyl acetate over a period of 360 s (video 10 times faster).
- movie S2 (.avi format). Video showing the immersion of bicontinuous capsule above in deionized water (pH 5 to 6), depicting the pulsed release of nanoparticle clusters, over a time scale of 10,000 s (video 300 times faster).
- movie S3 (.avi format). As above, following immersion in deionized water with pH 9.35 over a time scale of 7000 s (video 200 times faster; air bubbles result from the immersion of the dried capsule in water and are not a result of dissolution).
- movie S4 (.mp4 format). Video depicting the mechanism and kinetics of solvent extraction of 1% (w/v) NaPSS + 0.3% (w/v) SWCNT aqueous droplet of ≃250 μm radius in neat ethyl acetate over a period of 250 s (video 25 times faster).
- movie S5 (.mp4 format). Video depicting the mechanism and kinetics of solvent extraction of 1% (w/v) NaPSS + 0.025% (w/v) Au nanoparticle aqueous droplet of ≃250 μm radius in neat ethyl acetate over a period of 250 s (video 10 times faster).

Supplementary Materials

note S1. Microfluidic device fabrication

Microfluidic devices were fabricated by frontal photopolymerization of a commercially-available multifunctional thiolene prepolymer, employed as a negative photoresist. The photoresist was polymerized between pre-drilled glass plates, and selectively developed to yield a solvent-resistant microdevice. Specifically, photomasks were designed using AutoCAD and printed on a high resolution photographic film with 40,000 dpi resolution (microlithography services, UK). Glass slides (corning, (75 x 25 x 1) mm^3) were first cleaned with acetone and allowed to dry, and then inlet and outlet holes were drilled with a carbide tipped drill bit. Thin strips of acetate sheet (100 μm) were used as spacers between glass slides to define the channel height. The photomask was placed onto the glass sandwich and cured under a UV-A lamp (Spectroline) at 0.7 W/cm^2 for 20 s. The photomask blocks the microchannels from UV exposure while the other areas were polymerized. The uncured photoresist was washed out by injection of ethanol and acetone, leaving behind clear microchannels for operation. The device was postcured for 30 min without the photomask. Channels were rendered hydrophobic by treating with 10 wt% octadecytrichlorosilane (OTS) in toluene for one hour after which the device was placed in a convection oven for 24 hours at 110 °C. Finally, nanoports (N-333, Upchurch) were attached to the device using an epoxy adhesive (Araldite, Huntsman).

note S2. Phase Diagram of NaPSS/SiO_2/H_2O mixtures

The experimentally measured phase diagram for NaPSS/SiO_2/H_2O mixtures is shown in Figure S1, based on turbidity measurements and visual inspection of the ternary mixtures. For improved compositional accuracy, 40 ml of each composition was prepared. Single-phase homogeneous regions are indicated as 'miscible' while phase separated regions with two phase (solid-gas) and co-existing three phase 'solid-gas-liquid' compositions are indicated as 'immiscible'. All data presented in this work correspond to initial mixture compositions in the 'miscible' region.

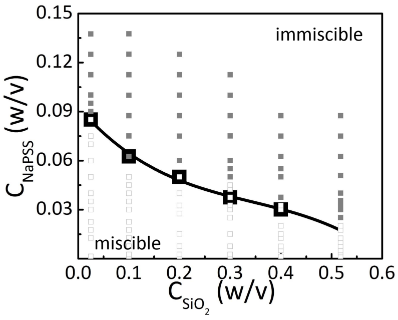

fig. S1. Phase diagram of NaPSS/SiO_2/H_2O mixtures. Phase diagram of NaPSS 70 kg/mol and 22 nm SiO_2 nanoparticles mixtures in water, showing one-phase ('miscible') and multi-phase ('immiscible') compositions.

note S3. Estimation of Péclet number

A comparison between the rates of reduction in droplet size and solute diffusion can be provided by the dimensionless Péclet number, which we write as $Pe = ER/D_0$ where E is the drying velocity, R is the droplet radius and D_0 is the diffusion coefficient. We estimate E from the initial dR/dt during isotropic reduction in droplet volume, and D_0 of the silica nanoparticle in the droplet using the Stokes-Einstein relation $D_0 = k_B T/6\pi\eta R$, taking the silica nanoparticle radius $R \approx 11$ nm and the viscosity η to be that of the ternary polymer/silica/H_2O mixture at temperature T; k_B is the Boltzmann constant. We find initial values of Pe between 3 - 20 in qualitative agreement with observed formation of capsule skin at early times and subsequent deformation of the capsule. Upon extraction, η evidently increases thus further increasing Pe, although such estimations are not expected to hold as the nanoparticle concentration increases, and upon agglomeration and demixing.

note S4. Additional analysis for data shown in main paper

In Figure S2 we show comparative $R(t)$ data for pure H_2O, 1 w/v% NaPSS (P), 10 w/v% silica (S), and 1 w/v% NaPSS + 10 w/v% droplets with initial droplet radius 140 μm. We find that as expected, the final capsule size, R_∞ depends on the solute content. We analyse the area, and deformation parameter (D= (R_{major} + R_{minor})/(R_{major} - R_{minor})) in Figure S3 for data shown in Figure 3A (main paper). Figure S4A is the corresponding $R_{minor}(t)$ plots for data shown in Figure 3B (main paper). At constant C_{SiO_2} (12 w/v%), an increase in C_{NaPSS} results in elongated capsules, as discussed in the main paper and shown in Figure S4B. While at constant C_{NaPSS} and $C_{SiO_2} \leq 0.5$ w/v%, deformed capsules are produced, an increase in C_{SiO_2} leads to a decrease in anisotropy yielding spherical capsules instead with dimples. Figure S5 shows the corresponding $R_{minor}(t)$ plots for data shown in Figure 3C (main paper). The inset in Figure S5 shows the dependence of capsule shell thickness on C_{SiO_2}. At $C_{SiO_2} \leq 0.5$ w/v%, capsules formed possess thin shells prone to deformation. Thick shelled capsules formed at high C_{SiO_2} (≥ 2 w/v%) resist deformation due to high bending and stretching energies, as discussed in the main paper. In Figure S6, we show time resolved R_{major}, R_{minor}, and deformation profiles for droplets with constant C_{SiO_2} of 5 w/v% (Figures S6A-C), and 10 w/v% (Figures S6D-F). At $C_{SiO_2} \leq 5$ w/v% and C_{NaPSS} 5 w/v%, dense capsules are formed and elongation is minimized. Deformation increases with C_{NaPSS} for $C_{SiO_2} \geq 10$ w/v%.

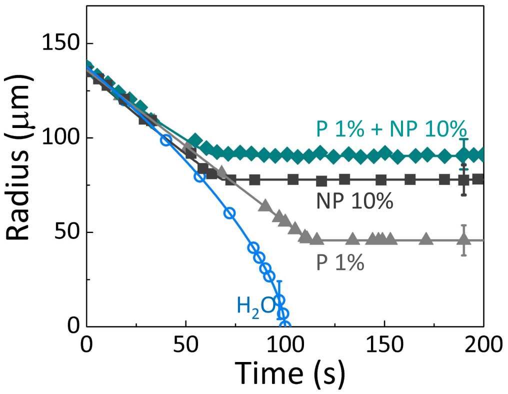

fig. S2. Kinetics of composite capsule formation for at initial C_{NaPSS}, 1 w/v%. Evolution of droplet radius with time during extraction for pure H_2O, 1 w/v% NaPSS (P), 10 w/v% silica (S), and 1 w/v% NaPSS + 10 w/v% SiO_2 droplets with initial droplet radius ≃140 μm.

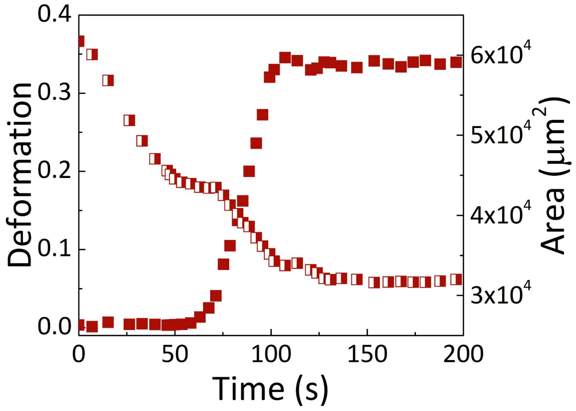

fig. S3. Deformation and area change of capsule obtained from 5 w/v% NaPSS + 12 w/v% silica droplet. Droplet extraction kinetics is analysed in terms of deformation and area for a droplet with high polymer and silica content (5 w/v% NaPSS + 12 w/v% SiO_2) with initial droplet radius ≃140 μm.

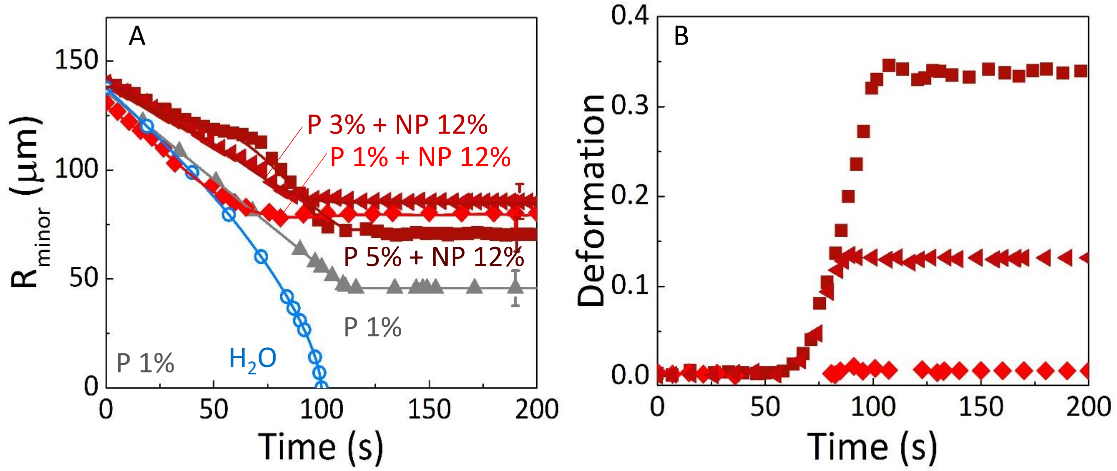

fig. S4. Kinetics of composite capsule formation for at initial C_{SiO_2}, 12 w/v%. (A) Corresponding minor axis R_{minor} data for Figure 2B (main paper) showing the effect of polymer concentration on extraction kinetics, at initial silica concentration, 12 w/v % of silica with initial droplet radius 140 μm; Pure H_2O and NaPSS 1 w/v% are included for reference (B). The increase in deformation with C_{NaPSS} is shown.

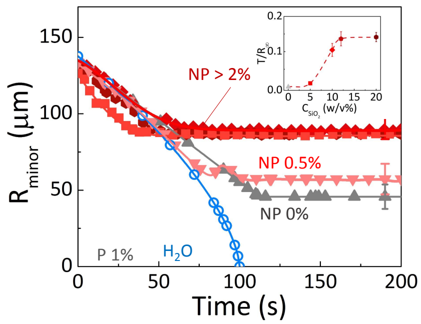

fig. S5. Corresponding minor axis R_{minor} data for Figure 2C (main paper). Effect of silica concentration on extraction kinetics at initial NaPSS (1 w/v%) concentration; results for NP of 2-20 w/v% overlap. The inset shows the dependence of the ratio of capsule shell thickness, T, to final capsule radius R_∞ on C_{SiO_2}.

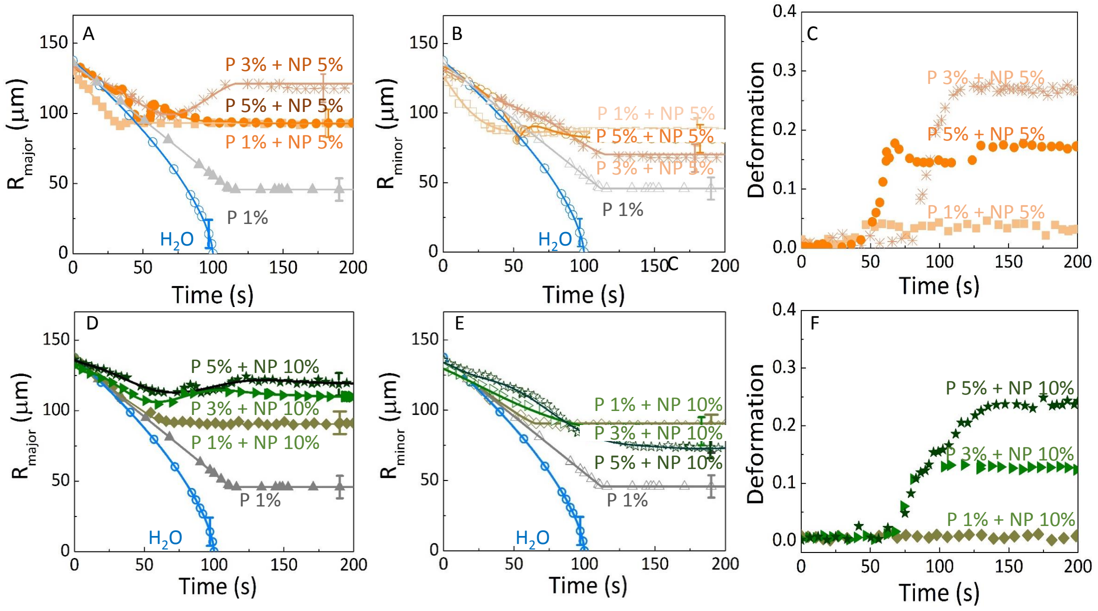

fig. S6. Evolution of droplet radius and deformation parameter with time during extraction for various NaPSS/silica composition. The effect of polymer concentration on extraction kinetics, at initial C_{SiO_2} 5 w/v % (A)-(C) and 10 w/v % of silica is shown in (D)-(F)respectively; Pure H_2O and NaPSS 1 w/v% are included for reference. R_{major} and R_{minor} are shown separately for clarity. The deformation parameter tracks the elongation of the capsules during the extraction process.

note S5. Additional SEM images of capsules

Additional SEM images illustrate the variety of anisotropic capsules that are attainable in our system for concentrations studied. External morphologies are shown in Figure S7 and internal microstructure in Figure S8 for selected compositions. Wide-view microscopy images of capsules obtained from specific compositions within the morphology diagram are shown in Figure S10 to demonstrate scalability and reproducibility of the capsule formation process. We take a horizontal line across the morphology map at C_{NaPSS}, 1 w/v% NaPSS (P) and show high resolution images of the capsule shell at varying C_{SiO_2} in Figure S9. The thickness of the capsule shell increases with increasing C_{SiO_2}.

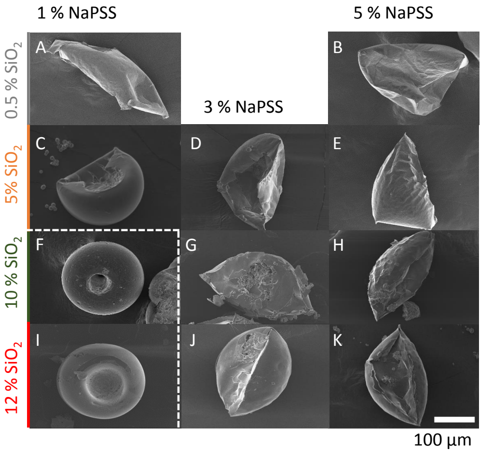

fig. S7. SEM showing the morphology of anisotropic composite NaPSS/silica capsule. At high C_{SiO_2}, $\geq$ 10 w/v %, and low C_{NaPSS}, (1 w/v %), dimpled capsules are obtained. Under all other conditions, non-spherical capsules with crumpled geometries are formed.

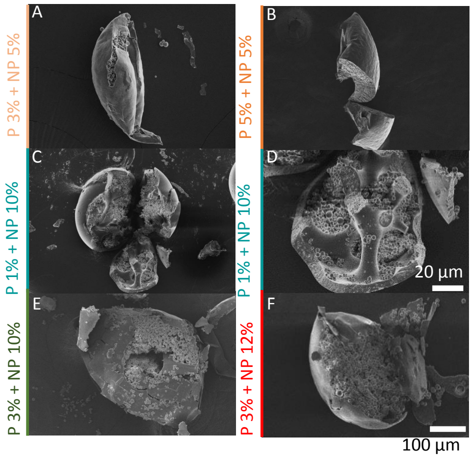

fig. S8. SEM images showing the morphology and internal microstructure of polymer-nanoparticle capsules as a function of NaPSS and SiO_2 content. (A) At low C_{NaPSS}, (3 w/v%), and C_{SiO_2} (5 w/v %), thin shelled capsules are formed (B) C_{NaPSS}, (5 w/v%), and C_{SiO_2} (5 w/v %) dense porous capsules are formed. The internal structure for bicontinuous capsules obtained from droplets with composition (C)-(D) 1 w/v% NaPSS + 10 w/v% SiO_2 and (E)-(F) 3 w/v% NaPSS + 10 w/v% SiO_2 and 3 w/v% NaPSS + 12 w/v% SiO_2 respectively.

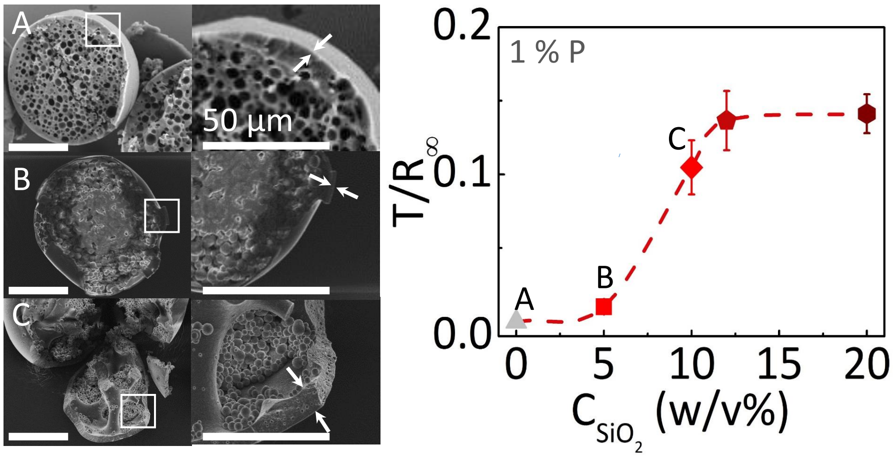

fig. S9. High magnification SEM images showing the internal morphology of neat polymer, and composite polymer-nanoparticle capsules as a function of NaPSS and SiO_2 content. Porous thin shelled spherical capsules are formed from neat polymer droplets with concentration (A) C_{NaPSS}, 3 w/v%, thin shelled capsules with largo cargo capacity for nanoparticle clusters are formed at (B) 1 w/v% NaPSS + 5 w/v% SiO_2 and bicontinuous capsules with internal scaffold structure is formed at 1 w/v% NaPSS + 5 w/v% SiO_2. (D) Dependence of shell thickness on nanoparticle concentration at initial C_{NaPSS}, 1 w/v%.

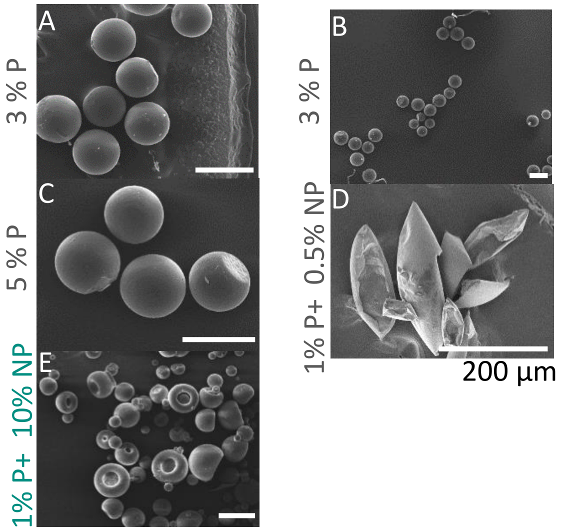

fig. S10. Wide-view SEM images showing neat polymer, and composite polymer-nanoparticle capsules as a function of NaPSS and SiO_2 content. Spherical capsules are formed from neat polymer droplets with concentration (A) - (B) C_{NaPSS}, 3 w/v%, (C) C_{NaPSS}, 5 w/v%. Ellipsoidal and dimpled capsules are obtained from composite polymer-nanoparticle droplets with composition (D) 1 w/v% NaPSS + 0.5 w/v% SiO_2 and (E) 1 w/v% NaPSS + 10 w/v% SiO_2 respectively. Capsules shown are obtained from droplets with R_o (A) - (C) 140 μm, (D) 100 μm - 250 μm and (E) 50 μm - 200 μm.

note S6. Energy dispersive x-ray spectroscopy of neat and composite NaPSS/silica capsules

To elucidate the spatial location of nanoparticle actives within the capsule we analyzed the capsules by energy dispersive x-ray spectroscopy. The data is shown in Figure S11. Neat polymer and nanoparticle capsules show only polymer and silica present respectively. The capsule shell, scaffold and nanoparticle clusters are analysed for composite capsules. A higher proportion of silica is observed to be present in the nanoparticle clusters compared to the capsule shell for anisotropic capsules, illustrated by a representative capsule produced from 3 w/v% NaPSS+ 10 w/v% initial droplet compositon. For bicontinuous capsules, the composition of the capsule shell and internal scaffold structure is found to be similar.

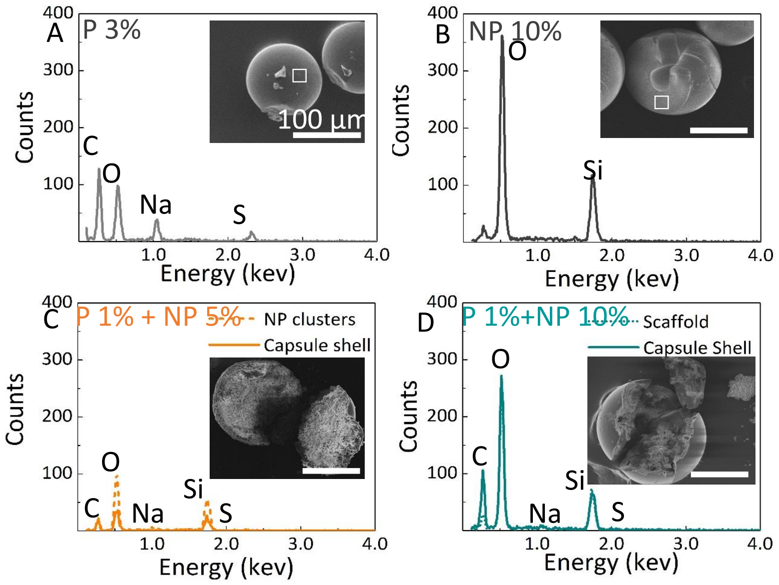

fig. S11. Energy dispersive x-ray spectrocopy (EDS) analysis of neat and composite NaPSS/silica capsules. EDS of (A) area (white square) of neat polymer capsule obtained from C_{NaPSS} 3 w/v% droplets showing the presence of only the NaPSS polymer. (B) area (white square) of neat nanoparticle capsule obtained from $C_{\mathrm{SiO_2}}$ 10 w/v%droplets showing the presence of only SiO_2. (C) representative area of capsule shell and nanoparticle clusters, of composite capsule with initial concentration 1 w/v% NaPSS+ 5 w/v% $\mathrm{SiO_2}$ showing a higher proportion of silica in the clusters compared to the capsule shell. (D) representative area of capsule shell and scaffold structure, of composite capsule with initial concentration 1 w/v% NaPSS+ 10 w/v% $\mathrm{SiO_2}$ showing a similar proportion of elements in the capsule scaffold and shell.

note S7. Analysis of dissolution of neat and composite NaPSS/silica capsules immersed in DI water (pH 5 - 6)

We have examined the dependence of the nanoparticle release mechanism on capsule morphology in Figure 5 of the main paper. In Figure S12, we show additional optical images of the dissolution of neat polymer capsules produced from 3 w/v% NaPSS initial composition, and the instantaneous release of nanoparticle clusters from a composite polymer-nanoparticle capsule produced from a droplet with 3 w/v% NaPSS + 10 w/v% SiO_2 initial composition.

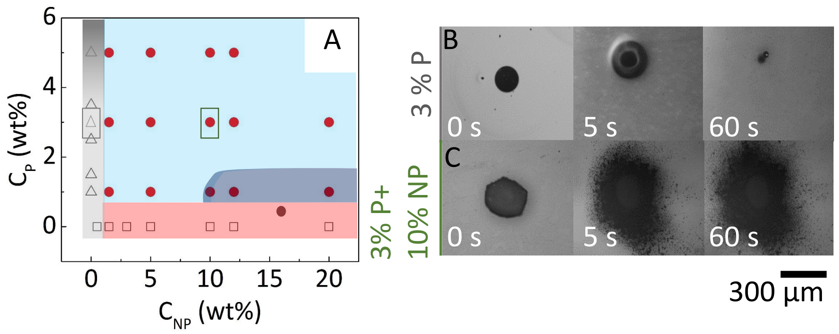

fig. S12. Dissolution of 3 w/v% NaPSS, and 3 w/v% NaPSS+ 10 w/v% SiO_2 capsules. (A)Phase diagram of capsule morphologies obtained from polymer-nanoparticle mixtures as a function of C_{NaPSS} and C_{SiO_2}. The empty grey and green squares represent initial droplet composition of 3 w/v% NaPSS, and 3 w/v% NaPSS+ 10 w/v% SiO_2 respectively. While (B) neat polymer capsules swell and dissolve over time (C) nanoparticle clusters are spontaneously released from composite polymer-nanoparticle capsule.

note S8. Spatio-temporal analysis of pulsed release of NP clusters from bicontinuous capsules immersed in DI water (pH 5 - 6)

We have examined the spatial distribution of release sites at the surface of a representative capsule, to resolve the number of active sites and any possible spatial correlation between them. We take as a reference bicontinuous capsules produced from droplets with 1 w/v% NaPSS + 10 w/v% SiO_2 and 1 w/v% NaPSS + 15 w/v% SiO_2 initial composition and final radius, $R\infty$, $\simeq$ 250 μm. These data correspond to Figure 5D of the main paper, and are obtained following immersion in DI H_2O, whose pH is 5-6. The frequency of these pulsed bursts is examined in Figure S9 for the three most active sites, indicated by the colored markers on a capsule produced from a droplet with 1 w/v% NaPSS + 10 w/v% SiO_2 initial composition is shown in Figure S13. The location of active release sites is graphically depicted in Figure S14 over three representative time intervals. Capsules are found to eject nanoparticle clusters from approximately 5-10 sites throughout their release process. These sites are found to be relatively uniformly distributed around the capsule surface. While some sites yield a single burst, others exhibit multiple bursts. The time interval between bursts evidently varies from site to site and over time, for a single site, however these occur between approximately 100 s to 1000 s intervals. We interpret these pulses as arising from the osmotic pressure build-up within the capsule and, possibly, the sequential rupture between internal compartments or pockets during release. The overall release profile is thus determined by the overlap of pulsed ejections from multiple sites over an exceptionally prolonged timescale, compared to those of capsules formed from the neat constituents. The dependence of the nanoparticle release intensity and release rate on the pH of the dissolution medium is shown in Figure S15 and Figure S16 for capsules produced from droplets with 1 w/v% NaPSS + 10 w/v% SiO_2 and 1 w/v% NaPSS + 15 w/v% SiO_2 initial composition and with final radius, $R\infty$, $\simeq$ 250 μm. At C_{SiO_2}, 15 w/v%, the release intensity is less sensitive to pH. Furthermore, no release is observed for composite capsules produced from droplets with 1 w/v% NaPSS + 20

w/v% SiO_2 initial composition. This is most likely because of irreversible aggregation of the silica nanoparticles at high comcentration.

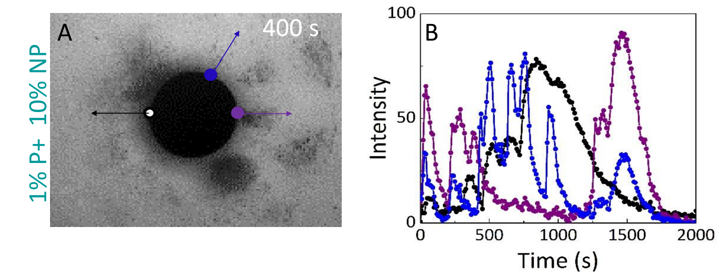

fig. S13. Pulsatile release of nanoparticle clusters from selected actives sites of a capsule obtained from 1 w/v% NaPSS + 10 w/v% SiO_2 initial droplet composition. (A) Optical image of a bicontinuous capsule 400 s after immersion in DI H_2O. The data correspond to Figure 5C of the main paper, for a capsule formed from 1 w/v% NaPSS + 10 w/v% SiO_2 initial droplet composition, and $R \infty 250 \mu m$. The markers indicate dominant release sites, and the colors correspond to the traces in (B), depicting the temporal release profiles and resolving the non-periodic nanoparticle cluster burst release.

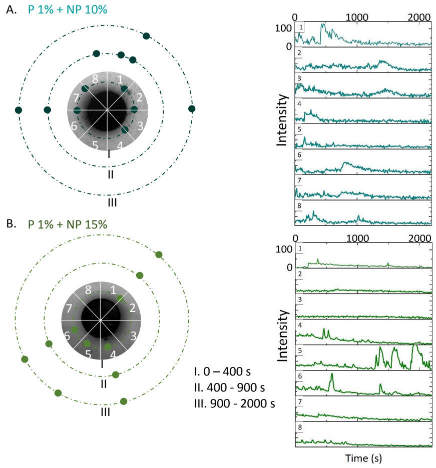

fig. S14. Pulsatile release of nanoparticle clusters from specific sectors of capsules obtained from 1 w/v% NaPSS + 10 w/v% SiO_2, and 1 w/v% NaPSS + 15 w/v% SiO_2 initial droplet composition. Active sites of cluster release on capsule surface at time intervals (I) 0-400 s, (II) 400-900 s, (III) 900-2000 s, indicated by each concentric circle with corresponding analysis of the release intensity from respective sectors of the composite capsule. Capsules shown are obtained from (A) 1 w/v% NaPSS + 10 w/v% SiO_2 and (B)1 w/v% NaPSS + 15 w/v% SiO_2 initial droplet composition, $R\infty 250\mu m$. Data shown are obtained from capsule immersion in DI H_2O. (A) is reproduced from Figure 5D of the main paper.

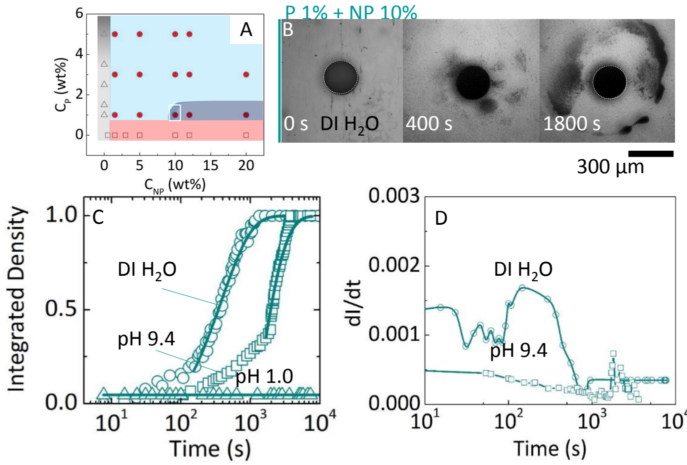

fig. S15. Analysis of release of nanoparticle clusters from a capsule obtained from 1 w/v% NaPSS + 10 w/v% SiO_2 initial droplet composition. (A) Phase diagram of capsule morphologies obtained from polymer-nanoparticle mixtures as a function of C_{NaPSS} and C_{SiO_2}, the white empty square represents initial droplet composition of 1 w/v% NaPSS + 10 w/v% SiO_2. (B) Optical images of dissolution of capsules of 1 w/v% NaPSS + 10w/v% SiO_2 at pH 5. The (C) release intensity and (D) release rate of the nanoparticle clusters from the bicontinuous scaffold is dependent on the pH of the dissolution medium.

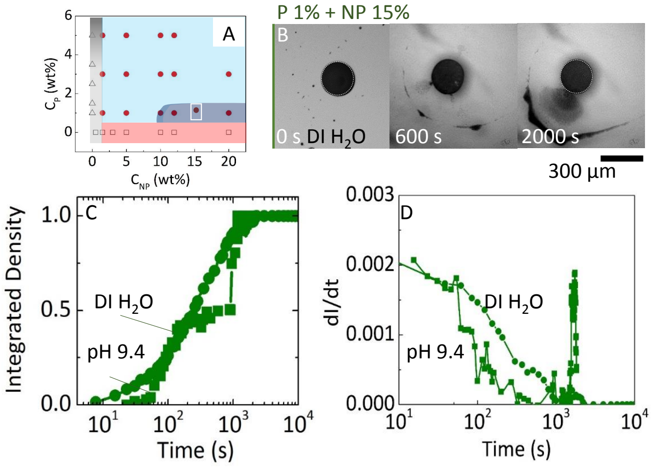

fig. S16. Analysis of release of nanoparticle clusters from a capsule obtained from 1 w/v% NaPSS + 15 w/v% SiO_2 initial droplet composition. (A) Phase diagram of capsule morphologies obtained from polymer-nanoparticle mixtures as a function of C_{NaPSS} and C_{SiO_2}, the white empty square represents initial droplet composition of 1 w/v% NaPSS + 15 w/v% SiO_2. (B) Optical images of dissolution of capsules of 1 w/v% NaPSS + 15w/v% SiO_2 at pH 5. The (C) release intensity and (D) release rate of the nanoparticle clusters from the bicontinuous scaffold is dependent on the pH of the dissolution medium.

note S9. Impact of payload type on composite capsule morphology and release profile

To investigate the generality of our approach, we demonstrate the encapsulation of nanofillers, single walled carbon (SWCNTs) nanotubes and Au nanoparticles, within polymer capsules. Optical images showing the time series of solvent extraction for droplets with composition 1 w/v% NaPSS + 0.3 w/v% SWCNTs and 1 w/v% NaPSS + 0.025 w/v% Au nanoparticles are shown in Figure S17A-B. As phase separation between polymer and nanofiller does not take place, unlike in the silica mixture, the receding liquid-liquid interface entraps the cargo and polymer phase inversion takes place as in the case of neat polymer. As a result, the resulting composite capsules remain largely spherical at the compositions investigated. The composite capsule microstructure is similar to that obtained from a neat polymer droplet (shown in SI Figure S9 and is not largely modified by the SWCNTs or Au nanoparticles, the payloads are therefore kinetically trapped within the polymer matrix. The internal microstructure of the composite capsules is shown in Figure S17C-D. The formation of bicontinuous internal morphologies evidently requires the crossing of the spinodal line along the solvent extraction pathway, which can be engineered to match the ternary solution thermodynamics as in the case of silica-polymer mixtures, but does not readily take place. Similarly, the formation of non-isotropic capsules requires the emergence of a (composite) stiff shell during the extraction process, which deforms – by bending, folding or crumpling – upon further volume reduction. The release of the entrapped SWCNT and Au payloads from the NaPSS/SWCNT and NaPSS/Au composite capsules is thus found to be mediated by the dissolution of the polymer matrix, which occurs within timescales of minutes instead many hours, as observed in SiO_2/NaPSS capsules, and shown in Figure S18.

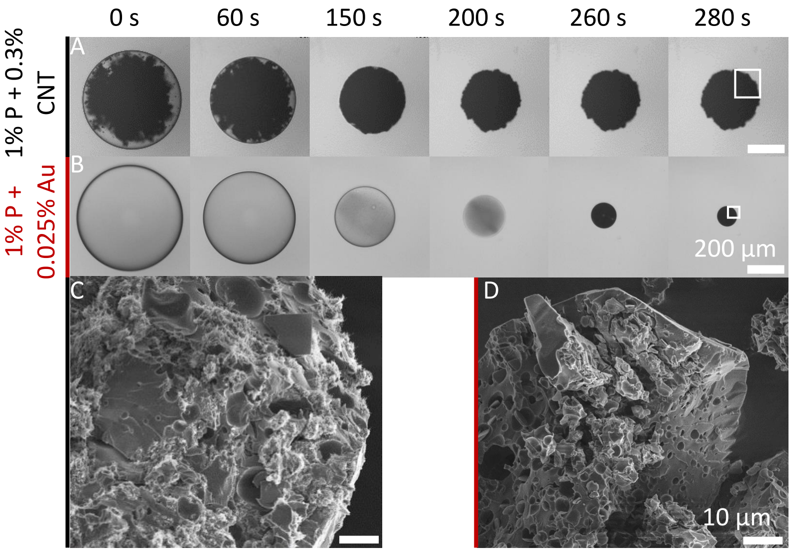

fig. S17. Formation and SEM images of internal microstructure of composite NaPSS/SWCNT and NaPSS/Au capsules. Optical images showing the time series of solvent extraction from droplets of (A) 1 w/v% NaPSS + 0.3 w/v% single walled carbon nanotubes (SWCNTs), (B) 1 w/v% NaPSS + 0.025 w/v% Au nanoparticles droplets; the initial droplet radius was $R_0 \simeq 250$ μm. Scanning electron microscopy images showing the internal microstructure of composite capsules obtained from droplets with 1 w/v% NaPSS and (C) 0.3 w/v% SWCNTs, (D) 0.025 w/v% Au nanoparticles.

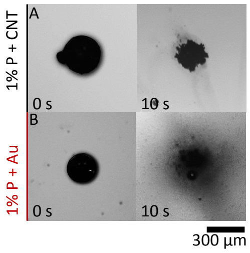

fig. S18. Release of SWCNT and Au nanoparticles from composite NaPSS/SWCNT and NaPSS/Au capsules. Optical images of dissolution of capsules fabricated from (A) 1 w/v% NaPSS + 0.3 w/v% single walled carbon nanotubes (B) 1 w/v% NaPSS + 0.025 w/v% Au nanoparticles, at pH 5.

Viscosity measurements of NaPSS/SiO_2/H_2O mixtures

table 1. Viscosity η [cP] of NaPSS/SiO_2/H_2O mixtures.

	C_{NaPSS} (w/v%)			
C_{SiO_2} (w/v%)	0	1	3	5
0	0	1.765	2.537	3.197
0.5	1.275	1.655	2.680	3.485
5.0	1.360	1.945	3.100	3.665
10.0	1.370	2.025	3.137	4.395
12.0	1.410	2.100	3.225	4.410
20.0	1.695	2.425	3.360	n/a
